# Suicide, Homicide, and Other Violent Deaths Among People Experiencing Homelessness in the United States: A Cross-sectional Study

**DOI:** 10.1177/00333549221087228

**Published:** 2022-04-25

**Authors:** Robert A. Kleinman, Nathaniel P. Morris

**Affiliations:** 1 Centre for Addiction and Mental Health, Toronto, ON, Canada; 2 Department of Psychiatry, University of Toronto, Toronto, ON, Canada; 3 Department of Psychiatry and Behavioral Sciences, University of California, San Francisco, San Francisco, CA, USA

**Keywords:** homelessness, suicide, homicide, violent death

## Abstract

**Objectives:**

Limited information exists about violent deaths among people experiencing homelessness (PEH) across the United States. Using data from a national reporting system, we describe characteristics of suicides, homicides, and other deaths classified as violent among PEH in the United States.

**Methods:**

We obtained data on demographic characteristics, mechanisms of injury, and circumstances surrounding violent deaths from January 1, 2016, through December 31, 2018, in 31 states from the National Violent Death Reporting System.

**Results:**

Of 122 113 violent deaths in 31 states during 2016-2018, 1757 (1.4%) occurred among PEH and 3952 (3.2%) occurred among people for whom homelessness status was unknown or missing. Of all violent deaths among PEH, 878 were suicides (1.1% of all suicides), 458 were homicides (1.6% of all homicides), 352 were of undetermined intent (2.8% of all deaths of undetermined intent), and 59 were the result of legal interventions (3.8% of all deaths due to legal interventions). Hanging/suffocation/strangulation was the most common mechanism of suicide among PEH (44.4%), followed by deaths due to firearms (21.6%). Firearms were the most common mechanism of homicide deaths among PEH (48.0%). Black PEH were more likely to die by homicide than by suicide, and White PEH were more likely to die by suicide than by homicide. Among the 843 suicide victims for whom additional information was known, 345 (40.9%) had a history of suicidal thoughts or plans, 245 (29.1%) had disclosed intent to die by suicide, and 183 (21.7%) were receiving treatment for a mental health condition.

**Conclusions:**

Efforts to reduce mortality and improve health outcomes among PEH should consider the high rates of violent deaths in this population.

As of 2020, more than 500 000 people were experiencing homelessness on a given night in the United States.^1^ People experiencing homelessness (PEH) tend to have higher rates of mental illness, substance use disorders, chronic medical conditions, infectious diseases, and victimization from violence than the general population.^2,3^ Poor health outcomes among PEH are driven by numerous factors, including extreme poverty, decreased and delayed access to medical care, inadequate treatment for mental illness and substance use disorders, lack of safety, and direct effects of homelessness, among others.^2,4,5^ Cohorts of PEH have been followed in several US cities and, compared with matched controls, have demonstrated higher rates of overall mortality, overdose deaths, and deaths due to homicide.^6-9^ A study of violent deaths among PEH in Maryland from 2003 to 2011 found that of 231 decedents in which circumstances of the death were known, 84 (36.4%) had an alcohol problem and 136 (58.9%) had another substance abuse problem at the time of death.^10^ Among the 270 decedents with blood alcohol testing results, 141 (52.2%) had detectable blood alcohol.^10^

Despite these findings, little is known about violent deaths among PEH in the United States. Surveys that assess the nationwide epidemiology of mental disorders, substance use disorders, suicidal behaviors, and criminal victimization (eg, National Survey on Drug Use and Health, National Crime Victimization Survey) often use household-based sampling methods that provide limited information about PEH.^11,12^ To improve understanding of health outcomes and mortality among PEH in the United States, this study used population-based surveillance data to characterize violent deaths among PEH in 31 states from January 1, 2016, through December 31, 2018.

## Methods

We obtained data on violent deaths among PEH from the National Violent Death Reporting System (NVDRS) Web-Based Injury Statistics Query and Reporting System (WISQARS).^13,14^ The most recent 3 years with available data spanned from January 1, 2016, through December 31, 2018, and 31 states (Alaska, Arizona, Colorado, Connecticut, Georgia, Illinois, Indiana, Iowa, Kansas, Kentucky, Maine, Maryland, Massachusetts, Michigan, Minnesota, New Hampshire, New Jersey, New Mexico, New York, North Carolina, Ohio, Oklahoma, Oregon, Pennsylvania, Rhode Island, South Carolina, Utah, Vermont, Virginia, Washington, and Wisconsin) had data available for all 3 years. NVDRS collects data on violent deaths from state-level reporting systems, aggregating information from death certificates, law enforcement, and medical examiner investigations, among other records.^15^ NVDRS defines a violent death as “a death that results from the intentional use of physical force or power, threatened or actual, against oneself, another person, or a group or community,” including suicides, homicides, deaths due to legal interventions, unintentional deaths by firearm, and deaths of undetermined intent.^15^ Individuals who died by suicide after being the suspected perpetrator of a homicide were included in a separate category (homicide followed by suicide) from other suicide victims and not included in the suicide counts in our study.^16^ A death due to legal intervention indicates an individual died as a result of contact with law enforcement personnel who were acting in the line of duty.^14^ A death with undetermined intent indicates that an individual died due to use of force in some manner but that the intent behind the death remained unclear.^15^ Other types of unintentional deaths (eg, unintentional overdose deaths), deaths due to war, legally sanctioned executions, and legal assisted suicides are not included.^15^ The NVDRS classifies a person as “homeless” if that person was residing in “places not designed for or ordinarily used as a regular sleeping accommodation for human beings,” shelter-based accommodations, or transitional housing for homeless people.^15^ The NVDRS WISQARS uses race categorizations (American Indian/Alaska Native, Asian and Pacific Islander, Black, White) and ethnicity (Hispanic, non-Hispanic, unknown) from the source documents, with individuals with multiple races recoded to a single race where possible using race-bridging methods.^15,16^

We analyzed demographic characteristics and mechanisms of injury of violent deaths descriptively, with violent deaths stratified by type (suicide, homicide, undetermined intent, or death due to legal intervention). Among violent deaths classified as suicides, we analyzed the circumstances related to the death.^15^ According to the NVDRS, information about these circumstances is derived from death certificates, medical examiner reports, police reports, and crime laboratories, among other sources.^15^

For deaths classified as homicides, we analyzed the relationship of the suspect to the victim. We conducted data analysis from April 16 to January 15, 2022. The Massachusetts General Brigham Institutional Review Board reviewed this study and determined that it did not meet criteria for human subjects research. The article follows the Strengthening the Reporting of Observational Studies in Epidemiology guidelines for cross-sectional studies.^17^

## Results

From January 1, 2016, through December 31, 2018, a total of 122 110 violent deaths occurred in the 31 states, of which 1757 (1.4%) were among PEH (male: 83.4%; female: 16.6%; White: 73.5%; Black: 18.2%; Hispanic: 11.3%). A total of 3952 violent deaths occurred among people for whom homelessness status was unknown or missing (3.2% of all violent deaths). Among PEH, 878 deaths were suicides (1.1% of all suicides), 458 were homicides (1.6% of all homicides), 352 were deaths of undetermined intent (2.8% of all deaths of undetermined intent), and 59 were deaths due to legal intervention (3.8% of all deaths due to legal interventions). Ten PEH died as a result of other causes of death classified as violent (unintentional firearm or suicide after being suspected perpetrator of homicide).

PEH accounted for 2.5% of all deaths by suicide and 5.0% of all deaths by homicide among American Indian/Alaska Native people; 1.7% of all deaths by suicide and 0.9% of all deaths by homicide among Black people; 1.1% of all deaths by suicide and 2.4% of all deaths by homicide among White people; 0.8% of all deaths by suicide among Asian/Pacific Islander people (data on deaths by homicide were suppressed); and 1.6% of all deaths by suicide and 2.1% of deaths by homicide among Hispanic people.

Suicide accounted for 878 of 1757 (50.0%) violent deaths among PEH (Table 1). Hanging/suffocation/strangulation was the most common mechanism of suicide (44.4%) among PEH, followed by deaths due to firearms (21.6%) and poisoning (13.7%). Among PEH who died by suicide where additional circumstances were known, 50.1% had a current mental health problem, 49.3% had a nonalcohol substance use problem, 40.9% had a history of suicidal thoughts or plans, and 27.9% had a history of suicide attempts (Table 2). In addition, 29.1% had disclosed suicidal thoughts or plans in the previous month, 21.7% were receiving current treatment for a mental health condition, and 38.9% had ever been treated for a mental health problem. Current or former military members accounted for 8.9% of suicides among PEH, representing 0.6% of total suicides among current or former military members.

**Table 1.** Demographic characteristics of people experiencing homelessness who were victims of violent deaths (N = 1757) and mechanisms of injury, 31 states, 2016-2018^a^

Characteristic	Suicide	Homicide	Undetermined	Legal intervention
Total	878 (100.0)	458 (100.0)	352 (100.0)	59 (100.0)
Year				
2016	279 (31.8)	132 (28.8)	141 (40.1)	21 (35.6)
2017	306 (34.9)	170 (37.1)	141 (40.1)	17 (28.8)
2018	293 (33.4)	156 (34.1)	70 (19.9)	21 (35.6)
Sex				
Female	146 (16.7)	63 (13.8)	79 (22.4)	—^b^
Male	732 (83.4)	395 (86.2)	273 (77.6)	—^c^
Race^d^				
American Indian/Alaska Native	36 (4.1)	31 (6.8)	13 (3.7)	—^b^
Asian and Pacific Islander	16 (1.8)	—^b^	—^b^	—^b^
Black	88 (10.0)	145 (31.7)	76 (21.6)	10 (16.9)
White	722 (82.2)	267 (58.3)	250 (71.0)	44 (74.6)
Hispanic origin				
Hispanic	75 (8.5)	74 (16.2)	36 (10.2)	10 (16.9)
Non-Hispanic	796 (90.7)	378 (82.5)	313 (88.9)	49 (83.1)
Unknown	—^b^	—^b^	—^b^	—^b^
Military status				
Current/former	78 (8.9)	30 (6.6)	22 (6.3)	—^b^
Nonmilitary	756 (86.1)	407 (88.9)	299 (84.9)	54 (91.5)
Unknown	44 (5.0)	21 (4.6)	31 (8.8)	—^b^
Age, y				
0-9	—^b^	—^b^	—^b^	—^b^
10-19	26 (3.0)	15 (3.3)	—^b^	—^b^
20-29	166 (18.9)	91 (19.9)	52 (14.8)	18 (30.5)
30-39	217 (24.7)	100 (21.8)	90 (25.6)	16 (27.1)
40-49	189 (21.5)	98 (21.4)	83 (23.6)	16 (27.1)
50-59	185 (21.1)	103 (22.5)	81 (23.0)	—^b^
60-69	80 (9.1)	45 (9.8)	38 (10.8)	—^b^
≥70	15 (1.7)	—^b^	—^b^	—^b^
Unknown	—^b^	—^b^	—^b^	—^b^
Mechanism				
Poisoning	120 (13.7)	—^b^	189 (53.7)	—^b^
Hanging/suffocation/strangulation	390 (44.4)	15 (3.3)	—^b^	—^b^
Firearm	190 (21.6)	220 (48.0)	—^b^	56 (94.9)
Motor vehicle	79 (9.0)	—^b^	14 (4.0)	—^b^
Fall	52 (5.9)	—^b^	12 (3.4)	—^b^
Drowning	22 (2.5)	—^b^	49 (13.9)	—^b^
Stab/cut	12 (1.4)	99 (21.6)	—^b^	—^b^
Struck by or against	—^b^	97 (21.2)	33 (9.4)	—^b^
Other (including fire)	12 (1.4)	10 (2.2)	13 (3.7)	—^b^
Unknown	—^b^	10 (2.2)	33 (9.4)	—^b^

a Data source: National Violent Death Reporting System.^16^ Data were available from 31 states for 2016-2018: Alaska, Arizona, Colorado, Connecticut, Georgia, Illinois, Indiana, Iowa, Kansas, Kentucky, Maine, Maryland, Massachusetts, Michigan, Minnesota, New Hampshire, New Jersey, New Mexico, New York, North Carolina, Ohio, Oklahoma, Oregon, Pennsylvania, Rhode Island, South Carolina, Utah, Vermont, Virginia, Washington, and Wisconsin. (Illinois and Pennsylvania provide data about at least 80% of violent deaths in the state.) Ten people experiencing homelessness died as a result of other causes of death classified as violent. All values are number (percentage).

b Reflects data that are suppressed due to counts <10.

c Data with counts ≥10 that have been suppressed to prevent ascertainment of counts <10 in other categories.

d Individuals may be included in >1 racial group or no racial groups.

**Table 2.** Leading circumstances in suicide deaths among people experiencing homelessness (PEH) where circumstances were known, 31 states, 2016-2018^a^

Circumstance involved in suicide death	No. (%)
Suicide deaths with known circumstances	843 (100.0)
Current mental health problem	422 (50.1)
Other [nonalcohol] substance abuse problem	416 (49.3)
History of suicidal thoughts or plans	345 (40.9)
Ever treated for mental health problem	328 (38.9)
Crisis in preceding or upcoming 2 weeks	269 (31.9)
Current depressed mood	255 (30.2)
Disclosed intent to die by suicide to another person within the previous month	245 (29.1)
Eviction or loss of home	244 (28.9)
Alcohol dependence	243 (28.8)
History of suicide attempts	235 (27.9)
Current treatment for mental illness^b^	183 (21.7)

a Data source: National Violent Death Reporting System.^16^ Data were available from 31 states for 2016-2018: Alaska, Arizona, Colorado, Connecticut, Georgia, Illinois, Indiana, Iowa, Kansas, Kentucky, Maine, Maryland, Massachusetts, Michigan, Minnesota, New Hampshire, New Jersey, New Mexico, New York, North Carolina, Ohio, Oklahoma, Oregon, Pennsylvania, Rhode Island, South Carolina, Utah, Vermont, Virginia, Washington, and Wisconsin. Data were from death certificates, medical examiner reports, police reports, crime laboratories, and other sources.^15^ Circumstances were classified as “known” in 843 of 878 (96.0%) suicide deaths among PEH.

b Current mental health treatment is defined in the National Violent Death Reporting System as “seeing a psychiatrist, psychologist, medical doctor, therapist, or other counselor (including religious or spiritual counselors) for a mental health or substance abuse problem; receiving a prescription for an antidepressant or other psychiatric medicine; attending anger management classes; residing in an inpatient, group home, or halfway house facility for mental health or substance abuse problems; or Alcohol or Narcotics Anonymous.”^15^

Among deaths by homicide by PEH, firearms were the most common mechanism of death (48.0%), followed by stabbing (21.6%) and being struck by or against something (21.2%) (Table 1). Firearms were the mechanism of assault in 60.0% of deaths by homicide where the victim was Black and 43.8% of deaths by homicide where the victim was White. For homicide deaths among PEH, the relationship of the suspect to the victim was most commonly unknown (35.6%), followed by friend/acquaintance (19.4%) and stranger (14.0%). Poisoning was the most common cause of deaths of undetermined intent (53.7%).

Black PEH were more likely to die by homicide (45.3%) of violent deaths among Black PEH) than by suicide (27.5%). In contrast, White PEH were more likely to die by suicide (55.9% of violent deaths among White PEH) than by homicide (20.7%). American Indian/Alaska Native and Hispanic PEH had similar rates of death by suicide and homicide.

## Discussion

By examining population-level data on violent deaths from 31 states, this study builds upon previous research on health-related outcomes among PEH. First, this study suggests that PEH in 31 states were disproportionately victims of violent deaths compared with the broader populations in those states. Point-in-time estimates from 2016 to 2018 indicate that 288 000 to 306 000 PEH lived in the 31 states, representing fewer than 0.2% of the combined population of those states^1^; however, PEH accounted for 1.1% of suicides, 1.6% of homicides, 2.8% of violent deaths of undetermined intent, and 3.8% of deaths due to legal interventions.

Second, suicide was the most common cause of violent death among PEH in 31 states from 2016 to 2018. Previous studies in Boston, San Francisco, and Maryland suggested that homicides were more common than suicides among PEH, although suicides were more common than homicides among a cohort of PEH in Toronto.^6,7,10^ The broad geographic coverage and different period of the current study, along with racial and other demographic differences among PEH in the involved studies, may account for these differences.

Third, although firearms were the most common cause of injury in suicides in the general population during the study period,^13^ strangulation/hanging/suffocation was the most common mechanism among PEH. These findings indicate that clinical focus on high-lethality means (eg, firearms, overdose prevention) may not fully address suicide risks among PEH and reinforce the need for more comprehensive suicide risk assessment and mitigation strategies.

Fourth, PEH appeared to be disproportionately likely to die from legal interventions. PEH often have high rates of police contact and may be disproportionately likely to be incarcerated compared with the general public.^18-20^ Further study is needed to understand the aspects of interactions between PEH and law enforcement officers that result in deaths among PEH and whether alternative crisis response strategies (eg, unarmed crisis intervention teams, verbal de-escalation) might reduce these risks and associated mortality among PEH.

Fifth, causes of violent death among PEH varied by race. Black PEH were more likely to die by homicide than by suicide, while White PEH were more likely to die by suicide than by homicide. These racial differences appear consistent with, although less pronounced than, broader US patterns in violent deaths. Among the general population, Black people are approximately 7 times more likely to die by homicide than by suicide, whereas White adults are approximately 5 times more likely to die by suicide than by homicide.^21^ The findings of our study highlight the need for further research into the associations between violent deaths among PEH and race and strategies to reduce race-related disparities. For example, PEH represented 5.0% of homicide deaths among American Indian/Alaska Native homicide victims, which was larger than other racial groups, and suggests the need for targeted interventions to reduce violence toward American Indian/Alaska Native PEH.

Finally, this study suggests that PEH who died by suicide were typically not receiving mental health services at the time of death. Identifying PEH at risk for suicide and providing appropriate interventions (eg, housing supports, mental health care, substance-related treatment) are critical public health tasks. Some screening prediction tools, such as the Mini International Neuropsychiatric Interview Suicidality subscale, have been studied and validated for potentially identifying PEH at highest risk for suicide.^22^ In addition, evidence-based strategies to engage PEH in mental health services should be broadly deployed. As examples, critical time intervention, where PEH are provided with intensive case management during transitions, has been found to reduce homelessness and negative symptoms among PEH with schizophrenia.^23^ Assertive community treatment, which involves a multidisciplinary treatment team with high staff-to-patient ratios and 24-hour availability providing in-community treatment, may also reduce homelessness and psychiatric symptom severity.^24^ Approximately half of PEH with known circumstances contributing to death by suicide had problems with substance use (other than alcohol), highlighting the need to target substance-related interventions toward PEH. While evidence-based treatments such as safety planning, cognitive behavioral therapy, dialectical behavioral therapy, and certain psychotropic medications exist for reducing suicide risk, few studies have examined these modalities among PEH.^25-28^ Further research is needed to determine the degree to which these interventions may be effective at reducing suicide rates among PEH and how to best implement these strategies.

### Limitations

This analysis had several limitations. First, although this study used data from 31 states during a 3-year period, these data may not be representative of the remaining US jurisdictions or other periods. Second, this study did not control for potential confounders, such as history related to poverty, education, incarceration, unemployment, or marital status, that may shape associations between homelessness and violent deaths. Third, housing status was unknown or missing for people representing 3.2% of violent deaths, potentially influencing comparisons between PEH and the general population. Fourth, classifying intent postmortem, such as determining the extent to which suicidal ideation contributed to overdose deaths, can be challenging.^29^ Lastly, retrospective determination of additional circumstances surrounding violent deaths, such as the existence of mental illness or involvement in mental health treatment, also introduces the risk of inaccuracies.

## Conclusions

Efforts to reduce mortality and improve health outcomes for PEH should consider the high burden of suicides, homicides, and other violent deaths among these populations. Because PEH face many challenges, including poverty, high rates of medical and psychiatric conditions, barriers to health care, and increased risks of victimization from violence, a comprehensive public health approach is necessary to achieve these aims. The findings from this study suggest a range of solutions, including wider development of housing supports, greater screening for suicide risks among PEH, expanded access to mental health and substance-related treatment services, means restriction policies (eg, limiting access to firearms or excess medication supplies), and overdose prevention strategies (eg, naloxone distribution), may be important for reducing violent deaths among PEH. Continued data collection and research on the health of PEH are essential for informing policy makers, clinicians, and others who seek to address this public health challenge.
